# Naturalness and Animal Welfare

**DOI:** 10.3390/ani8040053

**Published:** 2018-04-05

**Authors:** James Yeates

**Affiliations:** RSPCA Wilberforce Way, Southwater, Horsham, West Sussex RH13 9RS, UK; james.yeates@rspca.org.uk

**Keywords:** animal welfare, natural behaviour, naturalness, species-specific behaviour, wellbeing

## Abstract

**Simple Summary:**

Many people feel that we should ensure that animals have natural lives and can perform natural behaviours. However, it is unclear what exactly we mean by ‘natural’ and how we can assess it scientifically. We might use naturalness to highlight possible suffering that needs looking into, as a rule of thumb about what is good for animals, and to establish a threshold for what is acceptable or unacceptable in how we affect animals. We can assess animals’ naturalness in terms of how similar they are to their closest wild counterparts, both in scientific assessments and in decisions about how we care for animals.

**Abstract:**

Naturalness is considered important for animals, and is one criterion for assessing how we care for them. However, it is a vague and ambiguous term, which needs definition and assessments suitable for scientific and ethical questions. This paper makes a start on that aim. This paper differentiates the term from other related concepts, such as species-typical behaviour and wellbeing. It identifies contingent ways in which naturalness might be used, as: (i) prompts for further welfare assessment; (ii) a plausible hypothesis for what safeguards wellbeing; (iii) a threshold for what is acceptable; (iv) constraints on what improvements are unacceptable; and (v) demarcating what is not morally wrong, because of a lack of human agency. It then suggests an approach to evaluating animals’ behaviour that is quantitative, is based on reality, and which assesses naturalness by degrees. It proposes classing unaffected wild populations as natural by definition. Where animals might have been affected by humans, they should be compared to the closest population(s) of unaffected animals. This approach could allow us both to assess naturalness scientifically, and to make practical decisions about the behaviour of domestic animals.

## 1. Introduction

Naturalness is often considered important. ‘Natural behaviour’ (and its reciprocal, ‘unnatural behaviour’) is commonly suggested as a concept within animal welfare literature and a criterion by which to assess interventions on animals [1,2,3,4] as well as a concept to evaluate public perceptions of animal welfare [5,6]. Others may think of naturalness as a value in its own right, suggesting that animals should be allowed to live naturally not for their own benefit but because interference with nature is wrong. In either case, the (un)naturalness of animals’ lives provides a rhetorical device to generally challenge practices such as intensive farming, impoverished laboratory cages, and bare zoological enclosures, or to specifically promote desirable behaviours seen in the wild.

However, natural behaviour is conceptually problematic and practically difficult to use objectively and consistently. There is a lack of an agreed definition or operational approach to assess how natural an animal’s behaviour is. It is a vague and ambiguous term, with the potential for, confusion, misuse, or misunderstanding. This is especially important because the (mis)use of the concept could lead us to dangerous conclusions. On the one hand, promoting naturalness might suggest that animals should not receive interventions such as unnatural analgesia or be kept by humans at all, or that animals should experience natural disease, predation and uncontrolled reproduction. On the other hand, any behaviour can arguably be described as natural in certain contexts (e.g., as natural responses to human interventions) and could be legitimised on that basis. Furthermore, there are evident conflicts between a concern to promote natural behaviour and other concerns for animals’ wellbeing that need resolution.

If animal welfare is an area of scientific enquiry, and natural behaviour is part of animal welfare, we should try to define it accurately and assess it scientifically. However, while a lot of excellent work has defined and evaluated behaviour in terms of function and feelings, surprisingly little has considered how natural behaviour might be defined and measured. Rather, people often make subjective judgments on whether an animal’s behaviour is natural [7,8]. Such subjective, qualitative judgements risk irrationalities, such as response biases, attentional selectivity, intransitivity, excessive focusing on certain traits, and inter- and intra-individual unreliability. In the absence of theoretical and empirical work, policy discussions can become confusing, misleading, or fruitless.

Defining and assessing natural behaviour may be difficult—like the rest of ethology and animal welfare science [9]. Indeed, rigorously assessing natural behaviour may ultimately prove too difficult. But the attempt is necessary.

This paper suggests an axiological approach that is amenable to scientific exploration, is based on reality rather than idealism, and allows us as humans to assess which interventions can allow animals to exhibit the greatest degree of natural behaviour. Rejecting, because of such conflicts, some intuitively popular assumptions on how natural behaviour relates to wellbeing, I want to nevertheless suggest ways in which natural behaviour can be useful to those concerned with animals’ feelings. Scientifically (or prescientifically), unnatural behaviour can prompt research questions, because they relate to matters that are important to animals in at least one context and, for similar reasons, natural behaviour can generate research hypotheses for testing. Ethically, the wellbeing of animals exhibiting natural behaviour can be used as a baseline of moral acceptability, and concern for (too much) unnatural behaviour can be used as a constraint on efforts to improve wellbeing.

This paper differentiates the term from other related concepts, and distinguishes naturalness and wellbeing as separate concepts. Nevertheless, it identifies contingent relationships in which the concern for natural behaviour can be used alongside the concern for wellbeing: (i) that unnatural behaviour might provide useful prompts for further welfare assessment; (ii) that natural behaviour might provide a rule of thumb in the complete absence of any other information in order to achieve a minimal level of wellbeing; (iii) that natural behaviour can be used to set thresholds for what is acceptable welfare (and vice versa); (iv) that avoiding unnatural behaviour might be used to set constraints on what welfare improvements are unacceptable; (v) that natural outcomes may be seen as amoral, through lack of (human) agency.

It then suggests an approach to evaluating animals’ behaviour that is quantitative, is based on reality rather than idealism, and allows us as humans to assess which interventions can allow animals to exhibit the greatest degree of natural behaviour. The first step is to identify whether the subject population is unaffected. If so, then this population can be assessed as natural by definition (thus avoiding paradoxical or absurd conclusions). If not, or if it is unclear, then the animal’s behaviour should be compared to the behaviour of the ancestral or most similar population of unaffected animals (e.g., wild members of the ancestral species). The quantification of the similarities and differences between these populations might represent the naturalness of the subject animals’ behaviour. This approach could allow us both to consider the concept of natural behaviour in a way that is amenable to scientific exploration, and to make practical decisions about the behaviour of domestic animals.

## 2. A Thought Experiment

Arguments about naturalness may seem relatively simple when considering some cases such as wild-caught animals kept in captivity, or behaviours that are never seen in wild populations. However, the concept may generate less obvious answers when considering more complex cases, in particular when some human activity has already (seemed to) engender an unnatural state.

For example, we might consider a brief thought-experiment about a new animal type: imagine a being created from genetically modifying a chicken so that it has no legs, no beak, no feathers. It does not peck, fly, dustbathe, or lay eggs. Genetically, this animal is very different from chickens, indeed the genetic differences are greater than those between animals we currently categorise as different species. Phenotypically, this animal has different morphology and behavioural patterns to all (other) chickens. Its creators call this animal a “Licken™”.

This raises a variety of questions. Are lickens unnatural? Are their features unnatural? What behaviour is to be expected of normal lickens? What aspects of their behaviour are natural and which are not—and how unnatural are they? What is the ideal for Lickens? What is a Licken’s essence? They were created by humans, not by direct divine creation, so is a Licken’s essence or purpose the purpose their creators envisaged? Should we ignore what chickens do, since lickens are genetically and phenotypically very different from chickens? Should we categorise lickens as a new species? If lickens are not chickens, on what grounds could we compare them to chickens? Might we still compare them to chickens from which they are derived (and compare chickens to their evolutionary ancestors)? Should we compare their behaviour to wild chickens, and if so, how? If there are no wild chickens, to what should we compare them? Should we compare them to red jungle fowl? Or should we compare them only to other lickens (which would presumably conclude that it is natural for lickens to have no legs, beak, sensation, feathers, or eyes and for them not to peck, fly, dustbathe or lay—and that we should resist any attempts to modify lickens so that they can peck etc). What would increase the naturalness of their behaviour? What should we when do we are faced with the choice to alter lickens so they either peck or dustbathe (but not do both)? Is creating lickens wrong? Is creating lickens against nature and do lickens, therefore have poor welfare?

## 3. Defining Animal Welfare and Natural Behaviour

To consider the relationships between these concepts, we need to know what we mean by them. ‘Natural’ has many meanings in different cases and is difficult to define [10,11], and, in general, it is what is (not) artificial or is touched by humans [12,13].

‘Animal welfare’ also has many meanings in different cases and is difficult to define [2,14], but a general idea of it relates to how an animal ‘attempts to cope with its environment’ [15]. This includes some consideration of its feelings [16] and its pathology, productivity or physiology [17,18,19]. To avoid confusion, I shall avoid the term welfare to relate to these concepts and instead use wellbeing. For this paper, wellbeing might be negatively defined as the aspects of welfare other than naturalness; more positively we might relate it to matters of animals’ feelings in the broad sense (including feelings due to physical, social and environmental factors.

## 4. A Good Definition of Naturalness

Before suggesting an approach, some theoretical work is useful to describe what we want from an approach to naturalness. We need an approach that is appropriate for scientific purposes, fitting with basic tenets of measurement theory and scientific method, particularly in treating naturalness as objectively observable and measurable. We also need an approach that provides sensible ethical guidance in practice, particularly in guiding decisions about animals in human care.

We need an approach that avoids making absurd conclusions, such as that different behaviour of unaffected wild animals are of unequal naturalness (i.e., it needs to conclude all unaffected wild animals’ behaviour is equally natural). We should similarly avoid concluding that the behaviours of any unaffected wild animals are unnatural (i.e., it needs to always conclude that their behaviour is natural) or, conversely, that any particular behaviour that never occurs is natural (i.e., it needs to conclude they are unnatural).

Four general questions are critical to help us work out an approach that meets our criteria:

Product or Production?—We could evaluate whether the causes of a behaviour (e.g., its genetic and ontogenetic causes) involve humans or not, or we could assess the product of such processes, in terms of mechanism, adaptive value, ontogeny, and phylogeny.

Real or Ideal?—Natural could relate to what really occurs (i.e., a wild-type) or to some idealistic concept that includes only certain desirable wild-type behaviours, for example excluding behaviours that indicate fear or pain (i.e., a sort of wild-archetype).

Point or Range?—Naturalness could describe a precisely, narrowly defined repertoire, or it could cover a range of behavioural repertoires. Most properties of animals’ behaviours are spread over a range (with the exception of behaviours that never occur).

Two-tone or Shades of Grey?—Behaviour could be described as more or less natural (i.e., a continuous property). But behaviour could also be categorised as natural versus unnatural (i.e., a binary categorical property), for example by setting thresholds on continuous properties.

Some answers, or combinations of answers, to these questions would be scientifically, ethically, or logically inappropriate. This process of elimination suggests that the appropriate combination is that naturalness is considered a real property, representing a range of behavioural repertoires and a matter of degree. This approach avoids implications that some unaffected wild animals’ behaviour are unnatural or less natural than others’, allows us to judge naturalness even in captive and domestic animals, and avoids admitting degrees of perfection. This approach also allows us to consider that wild animals’ behaviour is always natural, and allows us to assess behaviours that may have been affected by man, e.g., when we breed domestic animals.

We perhaps also want an account that fits, reflectively, with how we intuitively assess naturalness. Of course, this should not be definitive, but it is a good place to start. I would suggest that we might expect assessments of naturalness to (partly) follow Shepard’s Universal Law of Generalization, i.e., that the likelihood of a learned response, previously associated with a conditioned stimulus, to a given test stimulus, is inversely proportional to the similarity between that conditioned stimulus and a test stimulus [20,21]. This suggests that we subjectively judge an animal’s naturalness by comparing it to wild animals. Furthermore, we might make an additional assumption that such qualitative assessments can be reduced to multiple qualitative comparisons [22]. While neither assumption is vital for the proposal to work in practice, they bring it closer to how people actually think.

## 5. A Proposed Definition of Naturalness

It is important to isolate naturalness from other, associated concepts. In particular, it is important to distinguish natural from descriptions of other phenomena. For example, natural behavior is not the same as the behaviour that is normal for the species (e.g., in terms of species-typical behaviours or [23] concept of each animal’s telos), nor is it the same as the idealised good bits of nature restricted to particular natural behaviours that are considered desirable for other reasons (e.g., Bracke & Hopster’s [4] definition of natural behaviours as limited to pleasurable natural behaviours), nor is it what is confers the greatest fitness (as evolution is not necessarily an optimising process). Most importantly, the definition of natural behaviour should not introduce concepts that actually relate to another concern (e.g., descriptions of behavioural needs, the frustration of which can lead to suffering—what is important there is the avoidance of suffering, not the performance of natural behaviour per se).

In fact, one might suspect that many of the times that people use the term natural behaviour, they really mean another concept, and are actually concerned about something else. For example, stereotypies are perhaps an archetypical unnatural behaviour but are arguably better conceptualised in terms of feelings (e.g., frustration or repeated coping attempts) or function (e.g., central nervous system disturbance [24]). These other concepts may indeed, relate to natural behaviour, and further work is needed to explore those relationships, but they are not the same concept as that of natural behaviour as something valued in its own right.

A proposed definition of natural behaviour is as being ‘unaffected by man’. This can apply fairly obviously to unaffected wild animals’ behaviour, the behaviour of which is therefore natural. It can also apply to the behaviour of captive animals insofar as what behaviour would be natural for them is the behaviour that they would have expressed, were they unaffected. If a behaviour occurs in captivity that would have happened anyway occurs in captivity, then it is natural, even though the animal is captive. If, however, a behaviour is caused to be different or absent, then it is unnatural to that degree. In the anthropocene epoch, where human impacts are global and pervasive, some unnaturalness may be widespread. In one sense, humans are natural insofar as they come from the same origins as other species. However, to consider human activity and its effects as natural would effectively imply the philosopher’s sense of natural as relating to the real physical world (which would mean all animals are natural in any circumstance).

We can then, for any given subject animal’s behaviour, operationally define the naturalness of that behaviour as its homology to the behaviour of uninfluenced equivalent animals or, in everyday terms, state that how natural an animal’s behaviour is depends on how similar it is to that of equivalent unaffected wild animals.

## 6. Relating Naturalness and Wellbeing

### Clearing the Field: Rejecting Some Approaches

Let us first dismiss some ways in which one might relate wellbeing and natural behaviour. Firstly, we can dismiss the idea that natural behaviour is conceptually part of wellbeing: they are logically independent concepts. Some forms of aberrant behaviour may be defined as poor health, but in most cases behaviour is not the same as either feelings or function. Animate, insentient beings such as plants can exhibit natural behaviour (broadly defined) without experiencing any valenced feelings (see Barnard and Hurst’s response to such a similar argument against their natural-functional approaches to animal welfare [25]). We may also distinguish between a behaviour that is natural and one that is motivated in an individual [26]. Natural behaviour and wellbeing are different concepts.

Some authors have tried to conflate the two concepts of natural behaviour and wellbeing together by restricting the concept of natural to what is good in nature [27] and excluding what is bad. For example, Bracke and Hopster [4] defined natural behaviours as limited to pleasurable behaviours. However, this approach seems inconsistent with the meanings of the words: it seems wrong to consider earthquakes as unnatural. Such concept may well be useful, but it is not one of natural behaviour.

Perhaps we might consider the value of natural behaviour and wellbeing as part of some overarching value such as integrity, authenticity, or telos, of which some elements apply to sentient animals and others do not (while some might apply to sentient artefacts such as affective artificial intelligences, and others to natural insentient forms such as plants). But, in the absence of a convincing architecture of such a relationship in a way that has practical relevance, it seems sensible to consider them as conceptually separate values (or at least as distinct, top-level overarching elements within a very general idea such as goodness).

Secondly, we can also reject the idea that naturalness is consistently associated with better wellbeing. There is limited evidence that natural behaviour reliably either leads to or signifies better wellbeing, and some evidence that it does not, in comparison to some unnatural alternatives [6,9,28]. For example, animals are not expected to enjoy (opportunities to perform) sickness-behaviours, flight, or antagonistic behaviours [29,30,31] in comparison to the absence of the eliciting stimuli (i.e., disease, antagonistic conspecifics or predatory threats). Similarly, animals may find or show enjoyment in exhibiting unnatural affiliative interactions with their human caregivers [32].

The evolutionary adaptation responsible for natural behaviour is likely to have optimised survival and reproduction rather than wellbeing. Natural behaviour may sometimes be maladaptive (e.g., escape behavior) and unnatural behaviour may be adaptive (e.g., exhibiting vices in restrictive stabling may be a coping mechanism [33,34,35]. Natural motivations or needs may be better satisfied by unnatural behaviour (e.g., interactions with humans), especially where the motivation relates to consummatory elements, and/or animals demonstrate a high degree of behavioural plasticity and flexibility. It is impossible to determine a priori what natural behaviour improves wellbeing [36,37]. Indeed, where natural behaviour can be valued because of the wellbeing it causes or signifies, then it seems more parsimonious to consider whatever it is that actually has value: motivations, behavioural needs, avoiding suffering, etc.

## 7. The Value of Natural Behaviour

How, then, can we use natural behaviour when evaluating animal wellbeing? I can suggest several ways.

### 7.1. Research Questions: Unnatural Behaviour as a Cue for Investigation

Unnatural behaviour might be taken as a prompt for a more detailed animal welfare assessment. The observation of unnatural behaviour may give us a heads up to prompt further investigation to assess wellbeing. The presence of an unnatural behaviour can be considered to indicate different (potentially better or worse) wellbeing than wild animals experience. The presence of natural behaviour can be taken to indicate merely that there are anthropogenic processes in play, i.e., that we are having an effect and therefore have a duty to get it right [14].

Such further investigation in such cases can aim to determine whether the wellbeing of animals performing unnatural behaviour is better or worse (or neither) than that of animals performing a more natural behaviour. In some cases, it may be both: animals performing an unnatural behaviour may have worse wellbeing than some conspecifics’ who do not exhibit that behaviour, but better wellbeing than others’ who also do not. For example those performing stereotypies in certain conditions may have better welfare than animals not kept in those conditions (e.g., if the stereotypical behaviour is a response to stress) but worse welfare than animals kept in those environments that do not exhibit that behaviour (e.g., if the stereotypical response is a (partially) successful coping mechanism). The presence of unnatural behaviour does not help us to know how animals’ wellbeing has been affected—it can only generate a two-tailed hypothesis—but its observation makes it worth finding out which is true by considering other information.

### 7.2. Research Hypotheses: Natural Behaviour as a Rule of Thumb

While hypotheses can be generated from many sources of inspiration, it seems sensible to test hypotheses where there is a plausible chance of disconfirming the null hypothesis: i.e., hypotheses that might be true, for example from low-powered pilot studies. At the same time, often we lack the luxury of research data and need to make prima facie assessments of what will improve an animal’s wellbeing, for example about more unusual pets’ care. In both cases, there is an argument for cautiously considering natural behaviour as a guide to what to do. Essentially, the observations of wild animals’ behaviour can be used, as (a series of) observational large-scale preference tests and long-term survival studies. Behaviours that are exhibited naturally are motivated [38] and not (too) harmful [37] within the natural context in which they occur. For example, if seeking cover occurs in an animal’s natural ecosystem, then it seems likely that the animal will find seeking cover rewarding and conferring some survival benefit, and/or not doing so distressing and dangerous. Similarly, what an animal chooses to eat in the wild might be foods that it will find (reasonably) rewarding and (reasonably) healthy in other contexts.

Useful examples in this direction include the study of Latham and Mason [39] and D’Eath and Turner [40]. In this sense, this approach is similar to the classic ethological studies, such as those in the Edinburgh pig park [41] and similar work by Per Jensen and colleagues in Sweden (e.g., [42,43,44]), where showing modern breeds’ behaviour in semi-natural settings provides an additional set of complementary information. One risk of this approach is that ensuring natural behaviour seems likely to set a relatively unambitious level of wellbeing: one in which the animals suffer fear, disease, and competition, for example. Where we identify that the rule of thumb achieves such a low level, we are then in a position to move beyond the rule of thumb. This problem does not mean it cannot be useful as a rule of thumb—merely that it must be used only as a rule of thumb. Perhaps, however, there are other, better rules of thumb that achieve a higher level. Another limitation of this rule of thumb is that the value of natural behaviour may be (partly) context-specific. The value of escape may (largely) depend on the (perceived) presence of threats. The value of heat-seeking (at least partly) depends on the prior body temperature. Some natural behaviour may be motivated regardless of the actual context (e.g., vigilance, hiding), perhaps, again, especially appetitive behaviours. Others may be motivated in (nearly) all contexts (e.g., some form of food consumption).

Similarly, the value of natural behaviour may be (partly) individual-specific. Different individuals may have different needs and preferences, some of which might be associated with an unnatural behaviour. In particular, while many natural motivations are demonstrated by domestic animals in naturalistic settings [41,45,46] and in captive settings [47], differences in both phenotype and environment may mean that different motivations occur, and/or a, different behaviour is adaptive—hence natural behaviour can only ever be used as a rule of thumb. The rule of thumb of promoting natural behaviour might therefore be more reliable for more wild-type animals in more naturalistic contexts, whose breeding and ontogeny are more natural, and less useful for highly domesticated animals in anthropogenic contexts.

However, in all cases, the presence or absence of (un)natural behaviour is only a rule of thumb, and its implications can be defeated by new information. As a slight aside, this rule of thumb might be rhetorically useful if part of the appeal of natural behaviour as a value in animal welfare is because manmade environments are commonly seen as being harmful. Natural environments tend to be compared to harmful unnatural ones, associated with living conditions in modern intensive farming, zoological gardens, or laboratory settings. As such, the rule of thumb is less that natural tends to be good, but that anthropogenic tends to be harmful. Of course, if one completely rejects any causative connection between wellbeing and natural behaviour at all, then the latter cannot serve as a valid rule of thumb, not even inferentially.

### 7.3. Ethical Baselines: Natural Behaviour as a Threshold of Acceptability

As well as the empirical considerations, we might also make an ethical decision that, the wellbeing of animals that exhibit a natural behaviour is morally acceptable. On a small scale, it could be concluded that any individual natural behaviour is associated with an acceptable level of welfare (perhaps by considering all natural states to be acceptable). On a larger scale, it could be concluded that the wellbeing of animals that perform completely a natural behaviour (i.e., genuinely wild animals) is acceptable.

If either argument is accepted, then a further argument might apply this threshold to animals showing unnatural behavior, so that an animal’s wellbeing is deemed acceptable if it is at least as good as the wellbeing of animals showing natural behaviour. This would involve valuing situations that cause unnatural behaviour basis of a wellbeing assessment of both animals showing natural behaviour and those displaying the unnatural behaviour: providing the latter is no lower than the former, then that situation is also acceptable.

This could legitimise naturalistic space allowances (e.g., in nests or burrows) or thermal ranges, even when they risk unpleasant experiences or worse health. One practical difficulty in applying this argument is that it is difficult to determine what aspect of nature one tries to match—for example a cage should be at least as large as the home burrow, or perhaps the usual territory (although one easy answer is both).

A more worrying implication is that this would lead to, in itself, a very low threshold, given the amount of suffering with which natural behaviour can be associated. Where it is natural for animals to starve to death, suffer untreated disease, and be hunted regularly, these might seem unacceptable baselines (although this argument may underlie ethical claims that, since animals are killed and eaten by predators in the wild, it is acceptable for animals to be eaten by humans). Perhaps one could only use good natural behaviour to set the baseline, but (as for Bracke and Hopster [4]) there is no apparent theoretical basis for picking and choosing which aspects of the natural situation to use as our baseline.

Looking on a larger scale, we might consider that the average wellbeing of animals in captivity should be at least as good as the average wellbeing of animals in the wild. Outcomes such as natural morbidity or mortality rate may be considered acceptable if they are equivalent to, or better than, what occurs in nature. Again, this still seems a very low threshold when harms such as starvation, predation, or disease are naturally prevalent or intensely unpleasant.

### 7.4. Ethical Constraints: Unnatural Behaviour as a No-Go Area

Natural behaviour and animal wellbeing might be considered as separate concepts that constrain us in different ways—with each limiting how much we should pursue the other. For example, the breeding of insentient animals or blind chickens might be considered unacceptable, even when they lead to welfare improvements [48,49]. (Reciprocally, the concern for animal wellbeing might make us avoid certain natural behaviours, for example giving unnatural analgesia to reduce natural pain-related behaviours).

One disadvantage with using each concern to balance the other is working what degree of unnatural behaviour crosses the line in the sand, or how to balance the two concerns. That would require, presumably, some way of measuring naturalness and some way of making that assessment ethically, comparable with measurements of wellbeing. Alternatively, animal welfare could be defined so that the concept of animal welfare is relevant (or meaningful) only in unnatural situations. In particular, the scope of the meaningful application of the term animal welfare could be limited to situations resultant in human interaction. This approach would effectively mean that natural states are neither bad nor good welfare.

This approach has the disadvantage that it prevents us from speaking about the welfare of animals in natural situations, and it seems inconsistent to restrict the conversation to animals under human control. In particular, this approach would make nonsensical any idea of improving the welfare of a wild animal harmed by natural causes (e.g., surgery to fix a leg fracture from a falling tree) since we cannot speak of its prior welfare. This argument has the practical conclusion of suggesting that humans have a moral responsibility to act to improve animal welfare only in anthropogenic contexts.

### 7.5. Natural Behaviour as an Amoral Product

A fifth way to consider naturalness is as an amoral concept (at least, outside human morality). Either it is the responsibility of the divine creator (and therefore beyond human morality) or it is the result of natural forces with no genuine autonomous agency. Humans may act to prevent natural behaviour or, by omission, allow it to occur. But a natural event itself is amoral. By the same token, the outcome of that event is also non-ethical, such that any suffering caused or signified is not our fault.

This approach has the (practical) advantage of delimiting our sphere of concern, albeit with the caveats that there may be little animal behaviour that is genuinely unaffected by human activity, and that determining it is difficult. Thus, on this approach, the question of assessing when a behaviour is genuinely natural becomes a question of defining the borders of animal ethics.

## 8. Assessment of Natural Behaviour

This allows us to evaluate the naturalness of an animal’s behaviour by several steps (Table 1) that essentially involve identifying unaffected animals, characterising them, and then comparing the subject animals to that characterisation. In Step 1, we should identify whether the subject animals are unaffected. In some cases, this is a matter of knowing enough of the animal’s history to make a reasonable assumption that human intervention has had zero or negligible effect. For such animals, the work is now done with regard to evaluating naturalness (but far from done in terms of ethological study). Their behaviour can be assessed as natural by definition (and this therefore reliably and tautologically avoids their behaviour ever being absurdly deemed unnatural). In comparison, where we know or suspect there has been human influence, then we need to follow further steps. These steps are effectively efforts to assess the effect of human influence on the animal’s behaviour by comparing its behaviour to evidence of what its behaviour would have been otherwise.

Step 2 involves identifying an appropriate, relevant, similar, and unaffected population for comparison. My suggestion is to compare such animals to a population (or populations) which (a) is assumed to be unaffected; (b) is the most similar to the subject animal; (c) there is sufficient scientific information about; (d) is relevant to the research question. In order to avoid missing natural behaviours, this population’s definition should have wide inclusion criteria (i.e., across a range of ages, genders, ecological niches, and seasons) unless there are good reasons to do so (e.g., comparing female gestatory behaviours of pandas in zoological collections to wild pregnant females, but their consummatory behaviour to all studied wild pandas).

In some cases, we can compare animals’ behaviour to their own behaviour prior to an interaction (e.g., prior to being captured) or to that of their immediate wild relatives (e.g., to uncaptured kin). In other cases, where the most similar unaffected population is not self-evident phylogenetically, we might need to perform steps 3–7 for several populations to identify the population that is most similar. In other cases, where there is no equivalent population to a subject population that meets criteria (a–d), then a comparison is not possible. This means that, in these cases, we simply cannot evaluate the naturalness of those animals’ behaviour (this does not mean that there is no such thing as natural behaviour for these animals—merely that we cannot confidently claim to know what it is).

This Step 2 cannot use comparisons with populations of affected animals. Studies of domestic animals in semi-natural conditions (e.g., [41,46]) can usefully demonstrate animals’ behavioural motivations in the absence of certain restrictions, but this does not prove that the observed behaviour observed is natural. Similarly, studies of feral populations descended from domestic animals can only show behaviours which may or may not be natural (although there might be an argument for using comparisons to feral animals that are reasonably believed to have reverted entirely to their wild-type state—an assessment which should be based on comparing those feral animals to definitely unaffected animals, using this process).

In Steps 3 and 4, we need to characterise the behaviour of both the comparison animals and the subject animals, in a way that conceptually includes all relevant behaviours of all unaffected animals of that population across all relevant contexts. This characterisation can legitimately generalise from representative samples, as a methodological practicality no different from other scientific inferences from specific samples to generalised natural laws, so long as these samples are thought to be representative. This representation should include the whole range of variation, including extreme outliers. It should avoid trying to characterise a subset of archetypical behaviours, as, otherwise, it risks concluding some unaffected wild animal’s behaviour to be unnatural.

Simple ethograms might classify the structures or consequences of the relevant behaviour as purely descriptive categories; more complex characterisations might quantify the latencies, durations, frequencies, magnitudes, intensities, inter-individual variation (for comparisons at the group level), etc as continuous data. In some cases, eliciting stimuli can also be described categorically or quantifiably. In order to avoid missing natural behaviours, the observations of these animals (where they are possible) should be performed over as long a period and of as many animals as possible, across an appropriate range of contexts (e.g., season) and animals (e.g., ages). Inevitably, restrictions of sampling will mean some behaviours are missed (as they might for any ethogrammatic effort), and this means we should be cautious before declaring a behaviour categorically unnatural.

Steps 5 and 6 involve comparing the similarity between the subject and comparator populations’ complete range of relevant behavioural repertoires. Ideally, no single behaviour should be evaluated in isolation, as all behaviours may have some degree of naturalness depending on their contexts, eliciting stimuli, and relationships with other prior and subsequent behaviour. There is perhaps always a most similar behaviour to which it can be compared (e.g., dolphin circus tricks might be somewhat similar to acrobatics in the high seas).

For simple quantitative comparisons, the *number* of qualitatively-similar behaviours that the subject animal shares with the unaffected animals might be scored positively, the number of differences negatively, and the results aggregated using (adapted versions of) conventional statistical analyses of similarity involving categorical data (e.g., based on Pearson’s chi-squared test or Tversky’s formula).

For more sophisticated comparisons, continuous traits of entire behaviour repertoires could be compared quantitatively in terms of their relative latencies etc and/or the similarities of the eliciting stimulus of each (e.g., food types), either by objectively quantifying the similarity between those stimuli (using a similar process to the steps 5–7) or by comparing their similarity from the animal’s point of view by quantifiably comparing the similarity of the animal’s responses to those stimuli in each case (e.g., hunting and consumption repertoires). To achieve some quantifiable commensurability, these comparisons might best be expressed as percentages of the unaffected behaviour’s characteristics.

**Figure 1:**
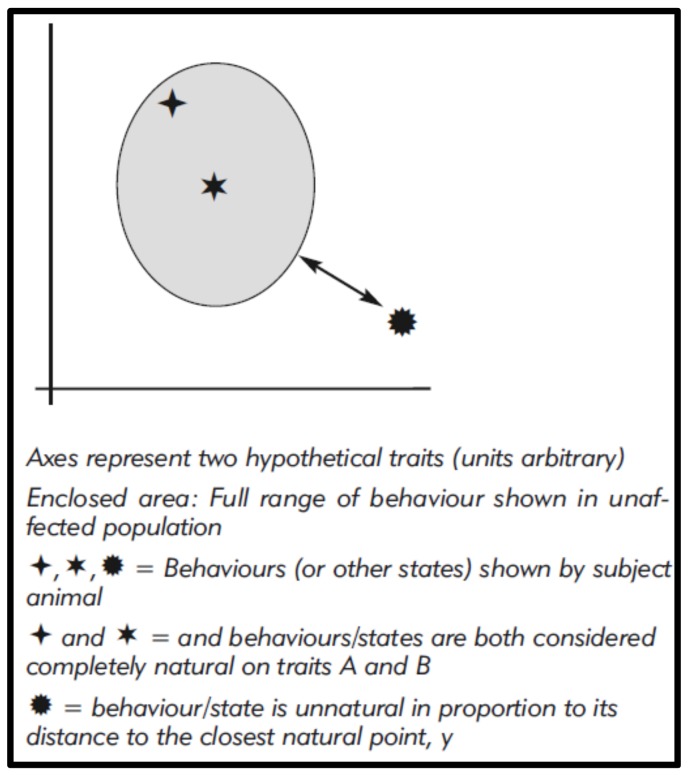
Quantitative assessment of behaviours relative to the behaviour of an unaffected population for two hypothetical traits.

These figures might even be modelled and, for visualisation, graphically represented using Cartesian methods (Figure 1), with unnaturalness, i.e., differences, corresponding to graphic distances [20,50]. This could also allow some weighting to reflect the perceived relative significance of differences (as we see for phylogenetic analysis), for example by giving greater importance to the complete absence of a common wild behaviour.

The resultant score provides a measure of the overall naturalness of the subject animals’ behavioural repertoire. We may then ethically choose to promote maximal naturalness (i.e., maximise the naturalness score) or to ensure that a certain threshold is met (e.g., ensure the naturalness score is above ‘*x*’ or that further human interventions should preserve or increase the naturalness score). These aims might need to be balanced with conflicting (or, in other cases, coinciding) objectives, such as avoiding suffering, improving biological function, or benefiting humans.

## 9. Examples

While an essay of this length cannot fully provide an in-depth evaluation of an animal’s behaviour, examples can demonstrate the process and its limitations. (These examples are not intended to generate any revolutionary conclusions about those animals but are for illustrative purposes).

1. Emperor Penguins (*Aptenodytes forsteri*) on a stable ice pack in Antarctica. Strictly speaking, ever since Johan Reinhold Foster and Captain Cook saw one there is the possibility that they are affected by human intervention but, unless they are disturbed by the assessors, this can probably be practically discounted, and these animals can be considered as being unaffected a priori, and thus their behaviour is natural. This example finishes at Step 2: there is no need for a comparative population as their behaviour is by definition natural. (Indeed, if one did such a comparison, they would be compared to themselves, and so be identical).

2. Emperor Penguins (*Aptenodytes forsteri*) on West Ice Shelf on the edge of Barrier Bay [51]. These appear likely to be a recent development [52] and, while colonies do shift sometimes, there is the reasonable possibility that these changes are due to human intervention. If so, we might evaluate how natural these penguins’ behaviour was by comparing them to unaffected conspecifics (e.g., [53]). In this case, this might give some circumstantial evidence to help answer whether the shift in the ecological niche is due to human effects—if the penguins are otherwise homogenous to definitely unaffected animals, then that might make us more confident to assume the difference is natural; if not, then that might make us think it is part of a suite of differences due to human effects.

3. Tigers (*Panthera tigris*) kept in a zoological collection and asked to perform some tricks. Comparisons to wild animals of the same subspecies would be more precise, but comparisons to all subspecies might be more appropriate if the information about their particular subspecies in the wild is sufficiently limited so that a restriction might hamper forming any valuable conclusions, so long as there are no know relevant differences between subspecies. Particular behaviours might be evaluated relative to wild animals. For example, captive animals might engage in more or less urine-spraying [54], or more stereotypic movement, for example 23–40% of time budget, depending on the enrichment provided [55], or similar travel (e.g., 14–22 km/day in wild populations [56] compared with 19 km/day in captive animals [57]). This assessment would necessarily be based on what has been studied in wild and subject populations; subsequent work (e.g., comparing other aspects of behaviour) might challenge or support earlier findings.

4. A domestic sow (*Sus scrofa dmesticus*) in a gestation crate. The ancestor of the domestic pigs, the Eurasian wildpig or wild boar (*Sus scrofa*) is existent in (to the best of our knowledge) unchanged form at least in some cases. There are a number of subspecies and geographical distributions but in the absence of particular reasons to focus on one, any behaviour observed in any unaffected subspecies and environment might be used for comparison in this case. This might use binary assessments, aggregated into an overall assessment. (Note the number generated here is useless on its own beyond noting that the behaviour is not perfectly natural and cannot be meaningfully compared to the tiger assessments).

5. A horse (*Equus ferus caballus*) used for riding and group-housed at pasture. One possible comparator population would be the extinct ancestors (perhaps the tarpan, *Equus ferus ferus*), relying on information from historical and paleontological sources (which to my knowledge is insufficient to generate any useful conclusions). Another might be extant relatives, but Przewalski’s horse (*Equus ferus prezewalskii*) is a reintroduced captive population (and so arguably affected) and there is a dearth of other extant caballoids. Any attempts to determine what is natural would seem so speculative that I would suggest we cannot make any significant conclusions about the naturalness of domestic horses’ behaviour.

6. A pet ferret (*Mustela putorius furo*). The most probable ancestors of the ferret are the European polecat (*Mustela putorius putorius*) and the Steppe polecat (*Mustela eversmanni*) [58,59,60], bred for more than 2–3000 years [61]. As such, perhaps the behaviour that is found in both ancestral species might be reasonably considered to be natural for pet ferrets (e.g., play [62]), but little more can be confidently ascribed, and it may be more cautious to conclude that it is again impossible (or meaningless) to determine what is natural behaviour for ferrets.

7. A genetically adapted *Fmr1-/y* mouse model (*Mus musculus*) for Fragile X syndrome [63]. Compared to wild-type mice without the modification, such mice may show significantly altered ultrasonic vocalization levels [64]; cognitive deficits in attention [65]; cognitive learning defects [66,67,68]; hyper-reactivity to certain sensory stimuli [69]; deficits in prepulse inhibition [70]; altered trace fear memory [71]; distances travelled during an open field test [72]; greater aversion to central mirrored chambers and fewer victories in a tube test of social dominance [73] and so on (see Kazdoba et al. 2014 for review [74]). However, these can only generate hypotheses about what might be natural, because the other comparator mice are evidently unnatural. Instead, such mice would need to be compared to behavioural measures of completely wild mice (and not just lab mice strains lacking a knock-out modification) in the wild (not in human environments such as elevated plus-mazes).

8. Lickens. Lickens might be compared to the remaining red jungle fowl (*Gallus gallus*) in South Asian forests. Red jungle fowl usually weigh less than 1 kg [75], are omnivorous [76] and devote considerable portions of their time budgets to foraging, even under unnatural environments [77]. Lickens clearly differ in these (and other) characteristics, and to that extent. In other words, we compare them to jungle fowl, not chickens or other lickens. Furthermore, lickens are almost identical to one another, compared to the heterogeneity of jungle fowl, i.e. lickens would show an unnaturally low level of variation at the flock level. Lickens do not show the form typical of species in general (i.e., a certain degree of pleomorphism), which indicates that the species is unnatural as a species (in addition to further attributions of unnaturalness at the individual level).

A comparison between lickens and chickens is therefore not directly relevant. However, when (tacitly) considering differences that make lickens further removed from the jungle fowl, then these comparisons may be a (short-hand) way of saying that lickens are more unnatural than chickens. Chickens have been domestically bred from within this species for centuries [78,79,80,81]. Compared to jungle fowl, domestic chickens (*Gallus gallus domesticus*) show some somewhat similar morphologies and behavioural responsiveness [82], but also show considerable differences in the actual behaviours shown in domestic contexts [83] and morphologies [84]. These similarities between domestic chickens and jungle fowl could be considered to imply they are natural; the differences could imply the former are unnatural. The differences between lickens and chickens, in which the former are less like jungle fowl would highlight that the modifications introduced in making lickens increase the progeny’s unnaturalness.

## 10. Implications, Controversies, and Further Work

Further theoretical and empirical work is needed to select between and refine the methodology sketched here and to work out the implications for particular species and populations.

Meanwhile, some potential implications can be predicted, and these predictions can be used to test the approach against our intuitions. In particular:

- No behaviour is completely unnatural as it will share some characteristic to some unaffected wild animals’ behaviour.
- No behaviour should be considered in isolation, but as a measure of animals’ overall behavioural repertoires.
- Natural behaviour can occur within unnatural contexts, if, and insofar as, it would (be expected to) have happened counterfactually (although it is unlikely that captive animals’ behaviour can be completely natural).
- Behaviour that involves humans may be somewhat natural (e.g., an animal avoiding a human as a predatory threat).
- Prior contact with humans does not in itself necessarily mean that an animal is then unnatural (but only insofar as it is affected by that contact).
- Humans can not only allow naturalness by non-interference but also increase it by counteracting additional interference (e.g., through enrichment, breeding programmes, rehabilitation etc).
- Sometimes we cannot say anything significant about the naturalness of an animal.

There is a lot of work to be done if this approach is to be used scientifically. In particular, the usefulness of the concept in scientific animal welfare assessments may be limited by predictable problems of validity (e.g., where we lack robust knowledge about some behaviours of the comparison population because of limited sampling), reliability (e.g., across studies that look at different subpopulations) and quantifiability (e.g., measurements of magnitude), at least in the medium term. It also seems likely that many initial characterisations of what is natural will be superseded as new data are generated. In particular, a rare natural behaviour may be initially deemed unnatural. There is nothing particular unusual about that, and this should prompt only science’s normal cautiousness about conclusions in the face of limited information.

This method of assessment might be complex and time-consuming—particularly, relative to rhetorical, sweeping statements on (un)naturalness. However, assessing the naturalness of an animal needs to be complex if we want it to be meaningful. Highlighting this complexity may deter people from using the terms lightly and unthinkingly (and may encourage them to use the terms for what they really mean, by ‘choice’). But, ultimately, if people are serious about valuing naturalness, then they need to be serious about evaluating it. Nature is complex. Behaviour is complex. Any robust consideration of natural behaviour can be expected to be complex too.

This method also calls on us to recognise our limitations. Where, for a given population, there is limited information about a comparison population’s unaffected behaviour (e.g., zoological specimens of a critically endangered species), or where it seems impossible to identify a comparison population at all (e.g., ferrets), then we may be unable to make any useful statements about the naturalness of that subject population with any confidence. Where we cannot say much about what is natural and, in those cases, we should simply remain silent and limit our talk to matters of wellbeing.

## 11. Conclusions

The concept of (un)naturalness captures common intuitive reactions of approval and repugnance, but it does not sit easily alongside a concern for animals’ feelings. The vague assumptions that naturalness is reliably associated with better wellbeing are unfounded. Consequently, those concerned with naturalness and those concerned with feelings may often find themselves in disagreement.

Nevertheless, for those concerned with feelings, naturalness can be useful in four ways: to prompt research questions, to generate research hypotheses for testing, as a baseline of moral acceptability, and as a constraint on ethical decisions. Combined, these approaches suggest that when we notice an unnatural state, we have a responsibility to ensure that we have not made those animals’ lives worse, by using other methods of assessing animal wellbeing such as pathology, physiology, and—without reference to what is natural—behavioural studies.

## Figures and Tables

**Table 1:** Steps for Assessment of Naturalness of Animal Behaviour.

No.	Questions		
1	Identify whether subject population is unaffected		
2	If subject population unaffected: Conclusion = subject population’s behaviour is natural	If subject population evidently affected: Identify most similar unaffected population(s) to subject population	If subject population possibly affected: Identify most similar unaffected population(s) to subject population
	Process complete	→ Step 3	
3	Characterise the (range of) behaviour within the unaffected population		
4	Characterise the (range of) behaviour within the assessed population		
5	For behaviours that are identical 5a. Identify behaviour in which the animal is exactly similar to the type as “natural” 5b. Weigh each trait 5c. Aggregate the weights for each trait by simple addition to generate an overall similarity score		
6	For behaviours that are not identical: 6a. Quantifiably describe each behavior 6b. Numerically score the degree of dissimilarity to the natural population for each trait to generate a dissimilarity score for that trait 6c. Weigh each trait 6d. Multiply the dissimilarity score for each trait by that trait’s weight 6e. Transform the dissimilarity scores into comparable units 6f. Aggregate dissimilarity scores into an overall dissimilarity score		
7	Subtract the overall dissimilarity score from the overall similarity score		
